# Inhibition of Polyamine Biosynthesis Reverses Ca^2+^ Channel Remodeling in Colon Cancer Cells

**DOI:** 10.3390/cancers11010083

**Published:** 2019-01-13

**Authors:** Lucía G. Gutiérrez, Miriam Hernández-Morales, Lucía Núñez, Carlos Villalobos

**Affiliations:** 1 Instituto de Biología y Genética Molecular (IBGM), Universidad de Valladolid, 47003 Valladolid, Spain; lgonzalezg@ibgm.uva.es (L.G.G.); nunezl@ibgm.uva.es (L.N.); 2 Instituto de Biología y Genética Molecular (IBGM), Consejo Superior de Investigaciones Científicas (CSIC), 47003 Valladolid, Spain

**Keywords:** colorectal cancer, DFMO, store-operated Ca^2+^ entry, store-operated currents, polyamines, TRPC1, sulindac

## Abstract

Store-operated Ca^2+^ entry (SOCE) is the most important Ca^2+^ entry pathway in non-excitable cells. Colorectal cancer (CRC) shows decreased Ca^2+^ store content and enhanced SOCE that correlate with cancer hallmarks and are associated to remodeling of store-operated channels (SOCs). Normal colonic cells display small, Ca^2+^-selective currents driven by Orai1 channels. In contrast, CRC cells display larger, non-selective currents driven by Orai1 and transient receptor potential canonical type 1 channels (TRPC1). Difluoromethylornithine (DFMO), a suicide inhibitor of ornithine decarboxylase (ODC), the limiting step in polyamine biosynthesis, strongly prevents CRC, particularly when combined with sulindac. We asked whether DFMO may reverse SOC remodeling in CRC. We found that CRC cells overexpress ODC and treatment with DFMO decreases cancer hallmarks including enhanced cell proliferation and apoptosis resistance. Consistently, DFMO enhances Ca^2+^ store content and decreases SOCE in CRC cells. Moreover, DFMO abolish selectively the TRPC1-dependent component of SOCs characteristic of CRC cells and this effect is reversed by the polyamine putrescine. Combination of DFMO and sulindac inhibit both SOC components and abolish SOCE in CRC cells. Finally, DFMO treatment inhibits expression of TRPC1 and stromal interaction protein 1 (STIM1) in CRC cells. These results suggest that polyamines contribute to Ca^2+^ channel remodeling in CRC, and DFMO may prevent CRC by reversing channel remodeling.

## 1. Introduction

Colorectal cancer (CRC) is the third most common cancer worldwide and the fourth most common cause of cancer death. There are about 1,250,000 new CRC cases and more than 600,000 related deaths every year [1]. CRC is considered a model of choice for cancer chemoprevention studies for two reasons: First, the molecular history of CRC is well established and involves early mutations in the adenomatous polyposis coli (APC) gene linked to hyperplasia followed by mutations in oncogenes and tumor repressors as in c-myc, K-ras, and p53 that lead to adenoma, adenocarcinoma, and carcinoma [2]. Second, colonoscopy enables monitoring and removal of benign polyps, thus, allowing the monitoring of the disease and assessing the efficiency of chemoprevention treatments.

CRC chemoprevention studies using cell lines, animal models, and even clinical trials which have provided multiple candidate compounds for chemoprevention. In top of most lists of cancer chemoprevention compounds is Difluoromethylornithine (DFMO), also named eflornithine [3,4]. DFMO is a suicide inhibitor of ornithine decarboxylase 1 (ODC1), the limiting step in the biosynthesis of polyamines by animal cells. Therefore, an excess of polyamine biosynthesis is involved in cancer, particularly in CRC. Consistently with this view, ODC is overexpressed in most CRC and other forms of cancer [4,5]. ODC polymorphisms have been also reported in CRC. In addition, there is strong correlation between specific polyamines and tissue growth, and different growth stimuli induce ODC activity. Moreover, a variety of tumor promoters induces ODC1 and tumor formation [4,5]. Metcalf et al. reported the synthesis of DFMO, a highly targeted drug whose mechanism involved irreversible inhibition of ODC1 [3]. DFMO inhibits colon carcinogenesis in rodent models including the *Apc*^Min/+^ mice with increased levels of ODC and polyamines in intestinal tissues. Administration of DFMO alone is effective in suppressing carcinogenesis in the small intestines of these mice. Combinations of DFMO and non-steroidal anti-inflammatory drugs (NSAIDs) are potent inhibitors of carcinogenesis in both the large and small intestines of these mice [5]. The grade of intestinal polyps is polyamine-dependent and the anti-carcinogenic effects can be rescued by putrescine. These findings support a role for polyamines in intestinal carcinogenesis in *Apc^Min/+^* mice. Moreover, in the last 10 years, several clinical trials indicate that DFMO may prevent CRC, particularly when provided in combination with NSAIDs, such as sulindac [6,7,8]. In fact, there is ongoing a large clinical trial, the S0820 Preventing Adenomas of the Colon with Eflornithine and Sulindac (PACES) trial, that is presently evaluating the effectiveness of the combination of eflornithine and sulindac in preventing colon adenomas that may change CRC chemoprevention [9]. However, in spite of the clinical relevance, the mechanisms by which polyamines affect cancer hallmarks and carcinogenesis remain to be established.

At the physiological level, polyamines have been involved in epithelial restitution, a process of transient activation of cell migration and/or proliferation after wounding for epithelial tissue repair. Recent data suggest that this process could be mediated by triggered Ca^2+^ influx operated by transient receptor potential channel 1 (TRPC1) and involves changes in expression of stromal interaction molecules STIM1 and STIM2 [10]. In addition, it has been shown that the caveolae protein caveolin1 [11], and the small guanosine-5’-triphosphate-binding protein RhoA [12], interact with and activates TRPC1 to stimulate rapid epithelial restitution after injury by inducing Ca^2+^ signaling. TRPC1 primarily functions as a cation non-selective channel within pathways controlling Ca^2+^ entry in response to cell surface receptor activation [13,14]. TRPC1, described for the first time in 1995 [13], was initially reported to be the ion channel involved in store-operated Ca^2+^ entry (SOCE), the most important Ca^2+^ entry pathway in non-excitable cells [15]. However, this view has been controversial since TRPC1 induces a non-selective cation current quite different from the small, Ca^2+^-release activated current (CRAC) that is very selective for Ca^2+^ first reported in mast cells [16]. This was solved in 2006 after the discovery of Orai1 channels [15]. At the molecular level, SOCE is activated after depletion of intracellular Ca^2+^ stores, a process sensed by the stromal interaction protein 1 (STIM1), that oligomerizes and interacts with Orai1 channels in the plasma membrane [17]. Now, the most extended view is that in some cells, SOCE is mediated solely by Orai1 channels while in others, TRPC1 may form ion channel complexes with Orai1 where TRPC1 tunes SOCE [18]. Interestingly, SOCE and molecular players involved in SOCE have been recently involved in carcinogenesis of CRC and other forms of cancer [19,20,21]. These data invite speculation on whether DFMO could prevent CRC acting on molecular players involved in SOCE.

We have recently reported that intracellular Ca^2+^ homeostasis is remodeled in CRC [22]. In short, CRC cells display enhanced SOCE and decreased Ca^2+^ store content relative to normal colonic cells and these changes contribute to cancer hallmarks, such as increased cell proliferation, cell invasion and resistance to apoptosis [22]. At the molecular level, enhanced SOCE is associated to increased expression of Orai1, STIM1, and TRPC1 in CRC cells, and decreased Ca^2+^ store content has been associated to decreased expression of STIM2 [22,23] and other genes involved in intracellular Ca^2+^ homeostasis [24]. Store-operated channels (SOCs) are quite different in normal and colon cancer cells. Specifically, normal colonic cells display typical CRAC like currents driven by Orai1 channels. These are very small, Ca^2+^-selective, inward rectifying currents. However, CRC cells display larger, non-selective currents with both inward and outward components that are mediated by both Orai1 and TRPC1 channels [22]. Interestingly, aspirin, and other NSAIDs including sulindac, that have been reported to prevent CRC [25,26], inhibit SOCE and cell proliferation and migration in CRC cells [27,28] suggesting SOCE molecular players as targets for cancer chemoprevention. Inhibition of SOCE and SOCs by salicylate (aspirin) and other NSAIDs is not direct. NSAIDs act as mild mitochondrial uncouplers, thus, promoting loss of mitochondrial potential, the driving force for mitochondrial Ca^2+^ uptake [27,28]. Accordingly, NSAIDs-induced defective mitochondrial Ca^2+^ uptake promotes Ca^2+^-dependent inactivation of SOCs and SOCE inhibition in CRC cells and other cell types [29,30]. Therefore, as SOCs are enhanced in CRC and modulated during epithelial restitution, we asked whether DFMO may influence Ca^2+^ channel remodeling in CRC.

Here we have investigated the expression of ODC in CRC cells and the effects of DFMO on Ca^2+^ channel remodeling in CRC cells. We found that ODC is overexpressed in CRC cells. In addition, we found that ODC suicide inhibitor DFMO inhibits expression of TRPC1 and STIM1 in CRC cells, thus, reversing Ca^2+^ channel remodeling in CRC and providing a mechanism for cancer chemoprevention by polyamine synthesis inhibition.

## 2. Results

### 2.1. ODC Is Overexpressed in CRC Cells

**Figure 1:**
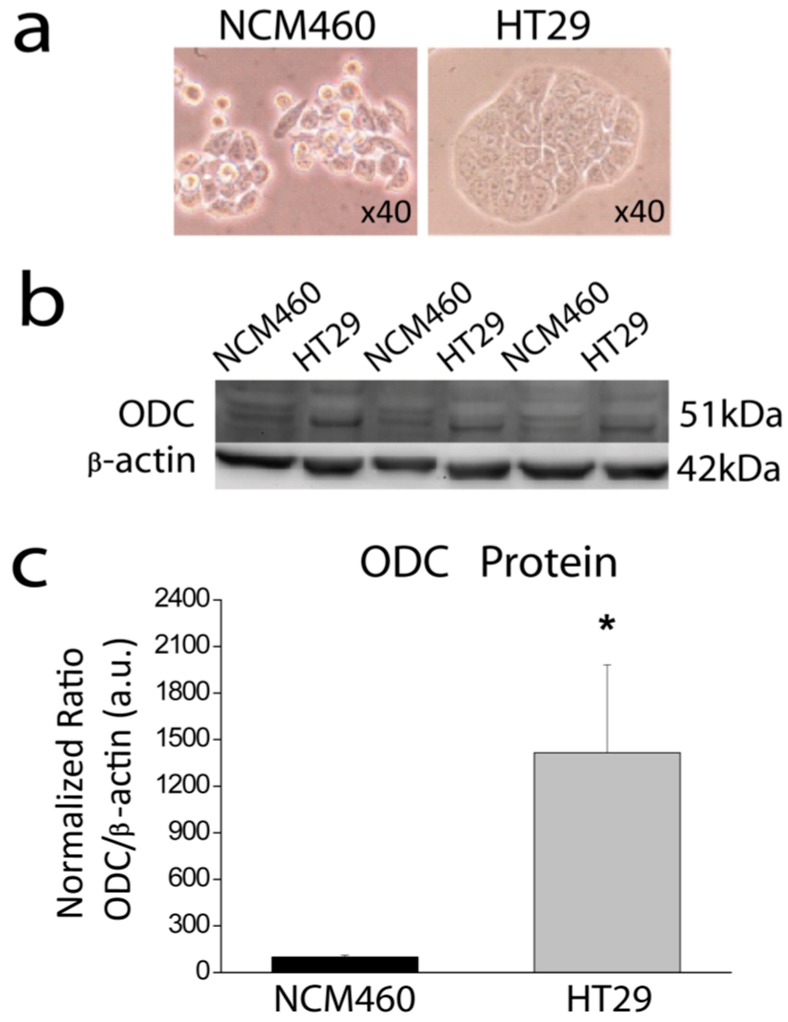
Ornithine decarboxylase (ODC) expression in normal colonic NCM460 cells and colon cancer HT29 cells. (**a**) Bright field images of human normal colonic NCM460 cells and human colon cancer HT29 cells. (**b**) Cells were lysed and subjected to Western blotting with anti-ODC antibody, followed by reprobing with anti-β-actin antibody for protein loading control. (**c**) Bar graphs represent ODC expression normalized to the β-actin content. Data are from *n* = 6 experiments (* *p* < 0.05).

It is well known that ODC is overexpressed in most CRC [4]. To assess whether this is also the case in our cell models we tested expression of ODC in cell extracts obtained from normal colonic NCM460 and colon cancer HT29 cells using Western blotting. Expression of β-actin was used as internal reference. Figure 1 shows that, as expected, expression of ODC is much larger in colon cancer HT29 cells than in normal colonic NCM460 cells (*n* = 6; * *p* < 0.05).

### 2.2. Effects of DFMO on Cell Proliferation and Apoptosis Resistance in HT29 Cells

**Figure 2:**
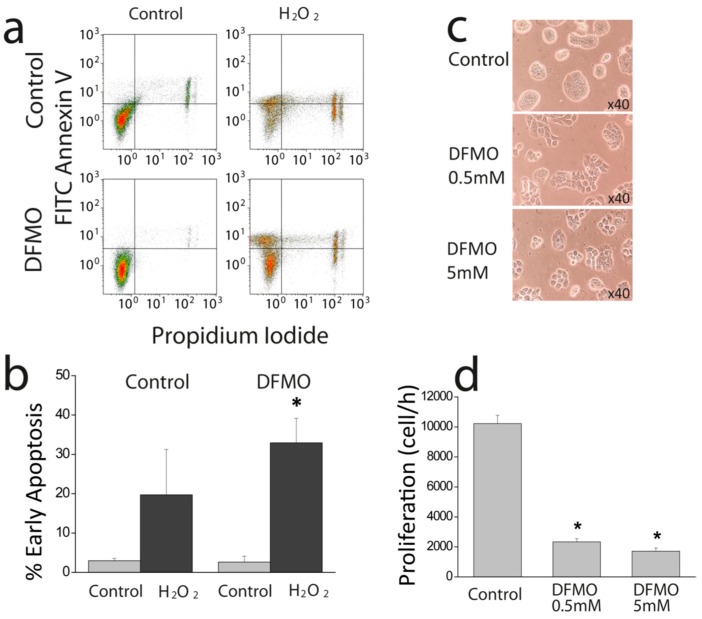
Effects of difluoromethylornithine (DFMO) on colorectal cancer (CRC) early cell apoptosis and cell proliferation. (**a**) Representative flow cytometry assay of fluorescein isothiocyanate (FITC) Annexin V (AV) and propidium iodide (PI) stained HT29 cells treated with vehicle or DFMO 5 mM, after treatment with solvent or H_2_O_2_ 2 mM for 150 min to test resistance to cell death. (**b**). Bars show percent of cells in early apoptosis in control and DFMO-treated HT29 cells before and after H_2_O_2_ (*n* = 3; * *p* < 0.05 vs. control). (**c**) Representative images of HT29 cells treated with vehicle or DFMO 0.5 and 5 mM. (**d**) Bars show rate of cell proliferation (Cells/h) of the same HT29 cells treated with vehicle or DFMO 0.5 and 5 mM. Data are from *n* = 3 independent experiments. * *p* < 0.05 vs. control.

DFMO has been reported to prevent colon cancer and cancer hallmarks [3,4,5,6,7,8,9]. To investigate whether this is also the case in our CRC cell model, we tested the effects of DFMO treatment on apoptosis and cell proliferation in HT29 cells. HT29 colon cancer cells were cultured in media containing solvent or DFMO. Then, cells were treated with solvent or H_2_O_2_ 2 mM for 150 min to test cell death and apoptosis using propidium iodide and Anexin V staining and flow cytometry. Figure 2a shows that H_2_O_2_ barely induces early apoptosis in colon cancer HT29 cells consistently with resistance to apoptosis characteristic of cancer cells. In contrast, in DFMO-treated cells, H_2_O_2_ induces a significant increase in early apoptosis indicating that DFMO reverses resistance to apoptosis in HT29 cells.

The effects of DFMO on HT29 cell proliferation were also tested. Figure 2c shows representative bright field images of HT29 cells treated with different concentrations of DFMO. Figure 2d shows that DFMO inhibits the rate of cell proliferation in colon cancer HT29 cells significantly. The effects are already large at 0.5 mM and do not increase at larger concentrations. These results indicate that DFMO treatment decreases cell proliferation and apoptosis resistance in colon cancer cells.

### 2.3. Effects of DFMO on SOCE and Ca^2+^ Store Content in HT29 Cells

**Figure 3:**
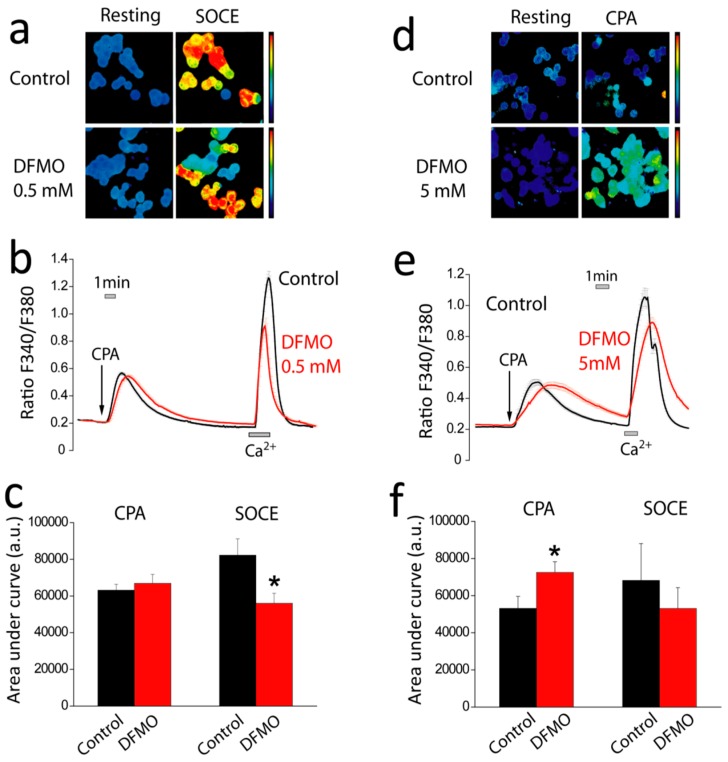
Effects of DFMO treatment on store-operated Ca^2+^ entry (SOCE) and Ca^2+^ store content in HT29 cells. (**a**) Representative ratio images of HT29 cells treated with vehicle (Control) or DFMO 0.5 mM before and after SOCE. (**b**) Representative calcium recordings of HT29 cells treated with vehicle (black) or DFMO 0.5 mM (red) during stimulation with CPA 10 μM in the absence of external Ca^2+^ (to stimate Ca^2+^ store content) and after extracellular Ca^2+^ addition (to estimate SOCE). (**c**) Bars show average ± SEM values of the rises in (Ca^2+^)_cyt_ measured as the area under the curve for both stimuli for control (black) and DFMO-treated (red) cells. (**d**) Representative ratio images of HT29 cells treated with vehicle or DFMO 5 mM during stimulation with CPA 10 μM. (**e**) Representative calcium recordings of HT29 cells treated with vehicle (black) or DFMO 5 mM (red) and stimulated with CPA followed by extracellular Ca^2+^. (**f**) Bars show average ± SEM values of (Ca^2+^)_cyt_ as in (**e**) representing Ca^2+^ store content in vehicle (black) and DFMO-treated (red) HT29 cells. Data are from *n* = 3 independent experiments. * *p* < 0.05.

Cell proliferation and resistance to apoptosis are related to SOCE and Ca^2+^ store content in CRC cells [22]. Accordingly, next, we tested the effects of DFMO treatment on both SOCE and Ca^2+^ store content, respectively. To this end, vehicle and DFMO treated cells were loaded with fura-2 and subjected to calcium imaging. Figure 3 shows representative fluorescence images of HT29 cells loaded with fura-2 before and after stimulation. Cells were stimulated with cyclopiazonic acid (CPA), a blocker of the sarcoplasmic and endoplasmic reticulum Ca^2+^ ATPase, in the absence of extracellular Ca^2+^ that induces the slow depletion of intracellular Ca^2+^ stores. The rise in intracellular free Ca^2+^ concentration ((Ca^2+^)_cyt_) induced by CPA is considered an estimate of intracellular Ca^2+^ store content. Once Ca^2+^ stores are depleted, cells are exposed to medium containing 1 mM Ca^2+^ to promote SOCE. The rise in (Ca^2+^)_cyt_ after Ca^2+^ re-addition, is considered a measure of SOCE [15]. Figure 3 shows representative recordings of HT29 cells treated with vehicle or DFMO and stimulated with CPA in Ca^2+^-free medium followed by stimulation with extracellular Ca^2+^. Figure 3 also shows the average values of the rises in (Ca^2+^)_cyt_ induced by both CPA and SOCE, respectively. We found that, at 0.5 mM, DFMO treatment has no effect on Ca^2+^ store content but inhibited SOCE significantly. However, at larger concentrations (5 mM) and larger incubation periods, DFMO also widens the rise in (Ca^2+^)_cyt_ induced by CPA in Ca^2+^ free medium consistently with increased Ca^2+^ store content and/or decreased Ca^2+^ clearance. In addition, we noticed that, in these conditions, the ability of DFMO to inhibit SOCE tended to decrease suggesting that the effects of DFMO on SOCE may be transient. These results suggest that DFMO treatment decreases SOCE and enhances Ca^2+^ store content, consistently with reversal of Ca^2+^ remodeling in CRC cells. SOCE.

### 2.4. Effects of DFMO on Store-Operated Currents in HT29 Cells

**Figure 4:**
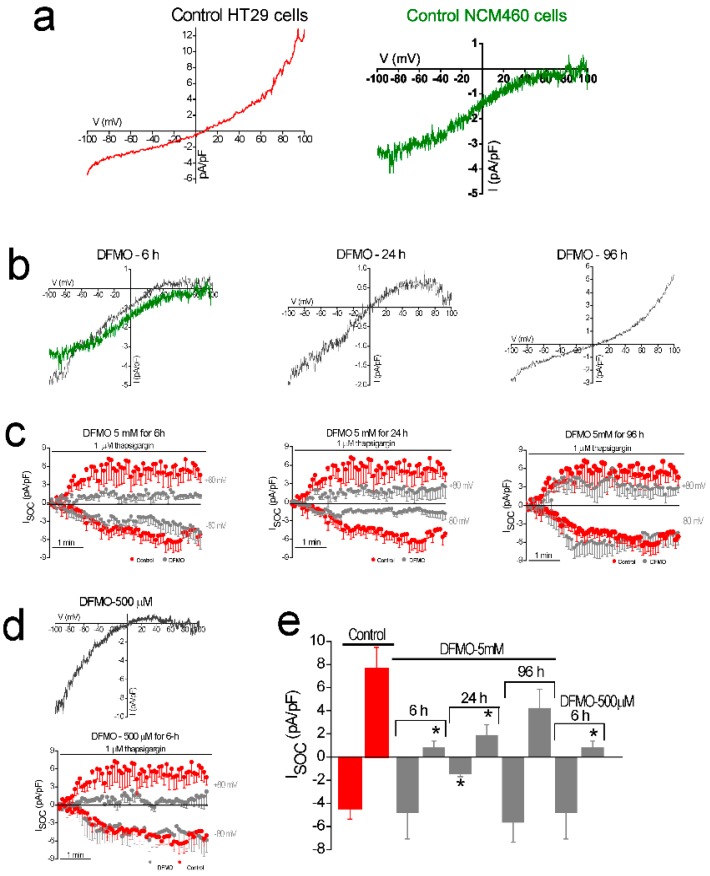
Effects of DFMO treatment on store-operated currents (I_SOC_) in colon cancer HT29 cells. (**a**) Current–voltage relationships (I–V) of I_SOC_ activated with 1 µM thapsigargin in HT29 colon cancer cells (Red) and NCM460 normal colonic cells (Green) with intracellular medium containing strong Ca^2+^ buffer (20 mM EGTA). (**b**) From left to right, I–V relationships for untreated HT29 cells (Red) and HT29 cells exposed to 5 mM DFMO for 6, 24, and 96 h, respectively. Shown in green is I–V relationship of representative current in NCM460 cells. (**c**) Averaged time course of I_SOC_ obtained from HT29 cells, at −80 mV and 80 mV, for control cells (red circles) and cells exposed to 5 mM DFMO for 6, 24, and 96 h, respectively (grey circles, mean ± SEM, *n* = 7–11). (**d**) Representative I-V relationship and time course of I_SOC_ in HT29 cells treated with 500 µM DFMO for 6 h (mean ± SEM, *n* = 7). (**e**) Bar graphs are averages of I_SOC_ measured after 5 min of stimulation with 1µM thapsigargin for control cells and DFMO-treated cells (mean ± SEM of 7–11 separate experiments, * *p* < 0.05).

We have studied the effects of DFMO treatment on store-operated currents (I_SOC_) in HT29 cells. Control and DFMO-treated HT29 cells were subjected to planar patch-clamp electrophysiology for I_SOC_ examination. I_SOC_ was activated with 1 µM thapsigargin with intracellular medium contained strong Ca^2+^ buffer (20 mM EGTA) to prevent Ca^2+^-dependent inactivation as reported previously [22]. Figure 4a–c show representative current–voltage (I–V) relationships in HT29 colon cancer cells (left) and NCM460 normal colonic cells (right). Figure 4b–c show representative I–V relationships and the averaged time-course of I_SOC_ in control and DFMO-treated HT29 cells. Treatment consisted of incubation of HT29 cells with 5mM DFMO for 6, 24, and 96 h. Next, DFMO was removed, and cells rested for 12 h before electrophysiological recordings. As previously described [22], untreated control HT29 cells displayed two different I–V profiles, a CRAC-like current and a mixture of a CRAC-like plus a nonselective I_SOC_ (Figure 4a, red recording). Therefore, the time course of I_SOC_ from control HT29 cells was comprised an inward and an outward component (Figure 4b).

Treatment of HT29 cells with 5 mM DFMO for 6 h abolished the outward component of I_SOC_ with no effect on the inward component (Figure 4a,b), producing a CRAC-like I–V profile similar to the currents recorded in normal colonic NCM460 cells [22]. However, in HT29 cells treated with 5 mM DFMO for 24 h the inhibition of the outward component was partial (Figure 4a,b). Additionally, in HT29 cells exposed to 5 mM DFMO for 96 h, the outward component was not inhibited. Interestingly, treatment of HT29 cells with lower concentrations of DFMO (500 µM) for 6 h inhibited the outward component of the I_SOC_ (Figure 4c). However, in those cells the I–V relationship reversed at 0 mV, corresponding to a non-selective I_SOC_. This suggests that the outward component was not completely inhibited, and the I–V relationship corresponds to a mix of a CRAC-like and a non-selective I_SOC_. Figure 4d shows average current amplitudes in control and DFMO-treated cells. These results suggest that DFMO inhibits the non-selective component, mostly outward, of I_SOC_ in HT29 cells and this effect is concentration- and time-dependent.

### 2.5. Putrescine Reverts SOCs Inhibition Induced by DFMO Treatment in HT29 Cells

**Figure 5:**
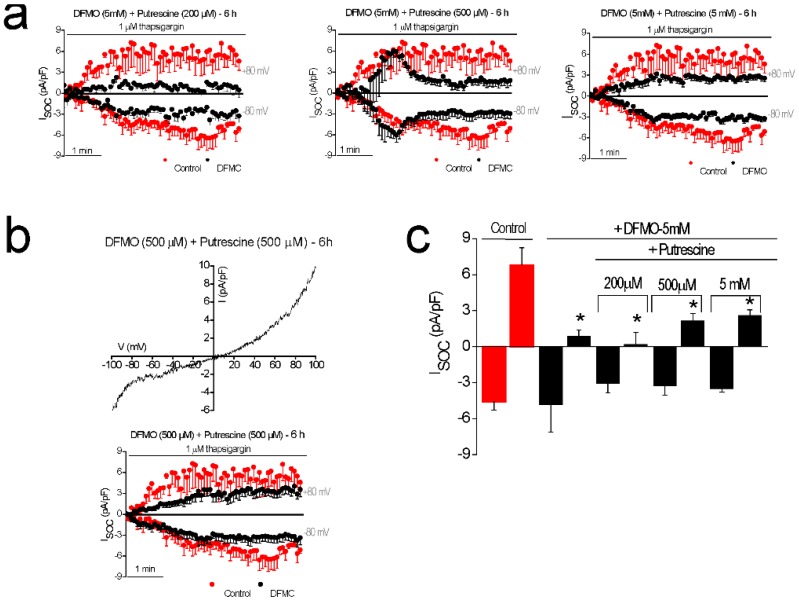
Putrescine reverses the effects of DFMO treatment on I_SOC_ in colon cancer HT29 cells. I_SOC_ were activated with 1 µM thapsigargin in HT29 cells with intracellular medium containing strong Ca^2+^ buffer (20 mM EGTA). (**a**) Averaged time course of I_SOC_ obtained from HT29 cells, at −80 mV and 80 mV, for control untreated cells (red circles) and cells exposed for 6 h to 5 mM DFMO plus 200 µM, 500 µM, and 5 mM putrescine, respectively (black circles, mean ± SEM, *n* = 10–12). (**b**) Representative I–V relationship and time course of I_SOC_ in HT29 cells treated for 6 h with 500 µM DFMO plus 500 µM putrescine (mean ± SEM, *n* = 10). (**c**) Bar graphs are averages of I_SOC_ measured after 5 min of stimulation with 1 µM thapsigargin for control cells and cells exposed to DFMO plus putrescine at different concentrations (mean ± SEM of 10 to 12 separate experiments, * *p* < 0.05).

To investigate whether DFMO effects on Isoc depend on ODC inhibition and polyamine depletion, next, we studied the effects of DFMO in the presence of the polyamine putrescine. To this end, Isoc were examined in control and DFMO plus putrescine-treated HT29 cells. Figure 5a shows time-course plots of averaged Isoc (± SEM) in DFMO-treated (5 mM, 6 h) cells in the presence of increasing concentrations of putrescine. At 200 μM, putrescine did not prevent the inhibitory effect of DFMO on Isoc. However, at larger concentrations, putrescine reversed the inhibitory effects of DFMO on the outward component of Isoc (Figure 5a).

Consistently, at equimolar concentrations (500 µM), putrescine fully reversed the effects of DFMO (Figure 5b). Figure 5c shows the average amplitude of Isoc in DFMO-treated cells in the absence and in the presence of different concentrations of putrescine. These results indicate that the reversal of channel remodeling by DFMO is due to its ability to inhibit polyamine biosynthesis.

### 2.6. Effects of DFMO on Expression of Genes Involved in SOCE in HT29 Cells

**Figure 6:**
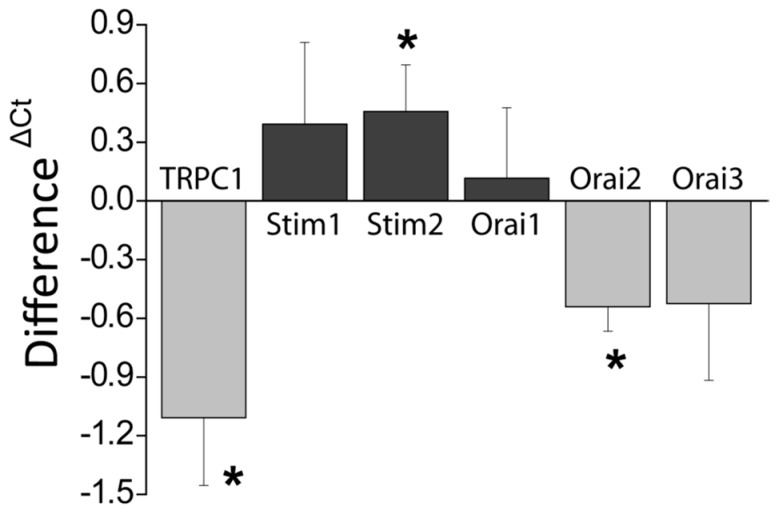
Effects of DFMO on the expression of genes coding for SOCE molecular players in HT29 cells. mRNA expression levels of selected genes were determined using qRT-PCR of extracts from control and DFMO-treated HT29 cells. β-actin was used as a reference. Data results are mean ± SEM from DFMO-treated cells relative to untreated cells Data are from *n* = 7, 7, 6, 6, 6, and 5 experiments for *TRPC1*, *STIM1*, *STIM2*, *ORAI1*, *ORAI2*, and *ORAI3*, respectively * *p* < 0.05.

Next, we have investigated the mechanisms of Isoc inhibition induced by DFMO treatment. To this end, mRNA expression levels of selected Ca^2+^ channels and sensors were determined using qRT-PCR in cell extracts from HT29 cells treated with vehicle (control) or DFMO. Results indicate that DFMO treatment decreases the expression of *TRPC1* and *ORAI2* genes significantly without affecting the expression of *ORAI1* and *ORAI3*. In contrast, DFMO increases expression of *STIM2* mRNA but not that of *STIM1* (Figure 6).

### 2.7. Effects of DFMO Treatment on Expression of Proteins Involved in SOCE in HT29 Cells

**Figure 7:**
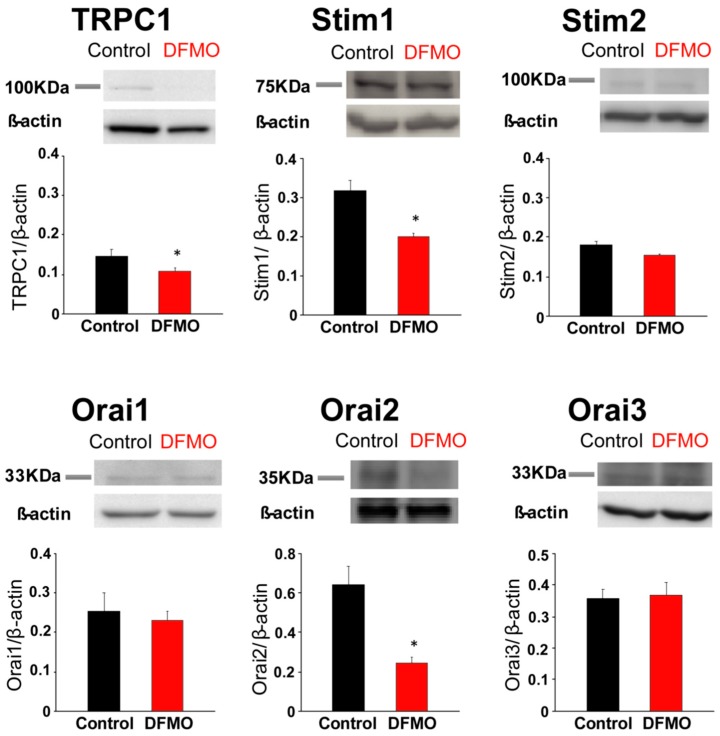
Effects of DFMO on the expression of proteins involved in SOCE in colon cancer HT29 cells. HT29 cells were treated with vehicle (control) or DFMO 5 mM, and then cells were lysed and subjected to Western blotting with antibodies against TRPC1, STIM1, STIM2, ORAI1, ORAI2 and ORAI3, followed by reprobing with anti-β-actin antibody for protein loading control. Bar graphs represent specific protein expression normalized to the β-actin content. Data are from *n* = 3 experiments (* *p* < 0.05).

We have also investigated the effects of DFMO treatment on expression of SOCE molecular players at the protein level. For this end, expression levels of the same selected Ca^2+^ channels and sensors involved in SOCE in CRC cells were determined using Western blotting of cell extracts from control and DFMO-treated HT29 cells. *β-actin* was used as a reference. Consistently with mRNA expression data, we found that DFMO treatment decreases expression of TRPC1 and ORAI2 proteins with no change in the levels of ORAI1 and ORAI3 (Figure 7). In contrast to mRNA data, STIM2 protein levels are not significantly affected in DFMO-treated cells. However, the levels of STIM1 proteins are decreased in DFMO-treated cells (Figure 7). As the expression of TRPC1 and STIM1 is enhanced in CRC cells relative to normal colonic cells [22], the present results indicate that DFMO treatment reverses, at least partially, the changes in expression of proteins involved in SOCE associated to CRC.

### 2.8. Effects of DFMO Treatment in Combination with Sulindac on Store-Operated Currents and SOCE in HT29 Cells

**Figure 8:**
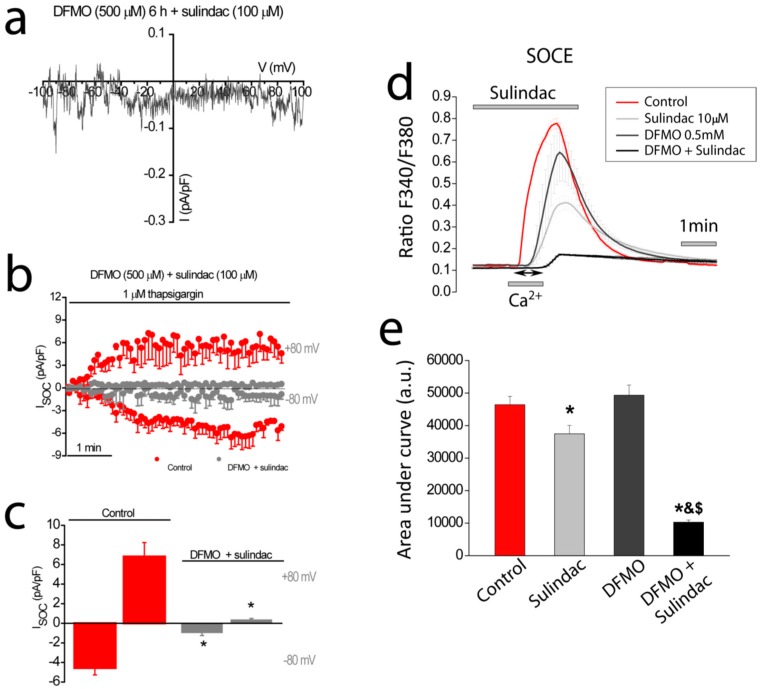
Effects of combination DFMO and sulindac on I_SOC_ and SOCE in HT29 cells. (**a**) I–V relationship and (**b**) averaged time course of I_SOC_ HT29 cells exposed to 500 µM DFMO plus 100 µM sulindac for 6 h (*n* = 4–12). (**c**) Bar graphs are averages of I_SOC_ measured after 5 min of stimulation with 1µM thapsigargin for control cells and cells exposed to DFMO plus sulindac (mean ± SEM of 4–12 separate experiments, * *p* < 0.05). (**d**) Average ratio recordings (mean ± SEM) of SOCE in fura-2-loaded cells treated with vehicle (Control), sulindac 10 μM, DFMO 0.5 M or the combination sulindac 10 μM plus DFMO 0.5 M. (**e**) bars are average (mean ± SEM) increases in (Ca^2+^) in at least three independent experiments. * *p* < 0.05 vs. control; ^&^ *p* < 0.05 vs. sulindac and ^$^ *p* < 0.05 vs. DFMO.

DFMO is considered one of the most promising candidates in CRC chemoprevention, particularly in combination with sulindac [3,4,5,6,7,8,9]. We have reported previously that sulindac promotes inactivation of the Ca^2+^-selective, inward component of Isoc in HT29 cells in a mitochondria-dependent manner. This effect is only observed in intracellular physiological buffer since it promotes Ca^+^-dependent inactivation [29]. Accordingly, next, we investigated the effects of the combination of DFMO and sulindac on Isoc in HT29 cells in conditions of intracellular physiological buffering. Figure 8a–c show that treatment with both DFMO and sulindac fully abolish both the inward and the outward components of Isoc. We also tested the effects of these compounds on SOCE. As shown in Figure 8d,e, low concentrations of sulindac and DFMO inhibit SOCE partially in HT29 cells. However, when added together, the combination of both compounds essentially abolished SOCE in HT29 cells.

## 3. Discussion

CRC is associated with the remodeling of intracellular Ca^2+^ homeostasis [20,23]. Specifically, we have reported recently that colon cancer cells display decreased Ca^2+^ store content and enhanced and modified SOCE [22], the most important Ca^2+^ entry pathway in non-excitable cells [15]. In addition, we showed that these changes contributed to cancer cell hallmarks including increased apoptosis resistance and enhanced migration and proliferation of cancer cells [22]. Here we show that DFMO, a suicide inhibitor of ODC, the limiting step in polyamine biosynthesis, previously reported to prevent CRC in vivo and in vitro, reverses this remodeling. These results highlight the role of Ca^2+^ remodeling in carcinogenesis, suggest that Ca^2+^ remodeling is promoted by excess polyamine biosynthesis, and finally, provide a mechanism of CRC chemoprevention by DFMO and its combination with sulindac.

First, we show that DFMO treatment increases the rise in (Ca^2+^)_cyt_ induced by CPA in Ca^2+^ free medium and enhances early apoptosis induced by oxidative stress. This effect may be explained by enhanced Ca^2+^ store content and/or defective Ca^2+^ clearance in DFMO-treated cells. However, as resting levels of (Ca^2+^)_cyt_ are not influenced by DFMO, the results suggest that DFMO modulates mainly Ca^2+^ store content. Ca^2+^ store content is related to the intrinsic pathway of apoptosis that involves mitochondria. In this pathway, mitochondrial Ca^2+^ overload induced by Ca^2+^ entry and/or release from intracellular stores favors mitochondrial permeability transition followed by cytochrome c release and activation of the apoptosome. We recently reported that colon cancer HT29 cells display decreased Ca^2+^ store content relative to normal colonic NCM460 cells [22] Consistently, HT29 cells were resistant to apoptosis induced by oxidative stress (H_2_O_2_) compared to normal cells [22]. Moreover, we also showed that decreasing Ca^2+^ store content in normal NCM460 colonic cells enhanced resistance to apoptosis induced by the same treatment. Therefore, it follows logically that the increased Ca^2+^ store content induced by DFMO may reverse this feature of the tumor cell phenotype, thus, decreasing resistance to apoptosis induced by H_2_O_2_. In addition, the low Ca^2+^ store content of tumor cells also favors activation of SOCE by physiological agonists that release only of a fraction the stored Ca^2+^ and promote a partial depletion of intracellular stores. Accordingly, the rise in Ca^2+^ store content induced by DFMO treatment may also constrain SOCE activation in tumor cells in physiological conditions.

**Figure 9:**
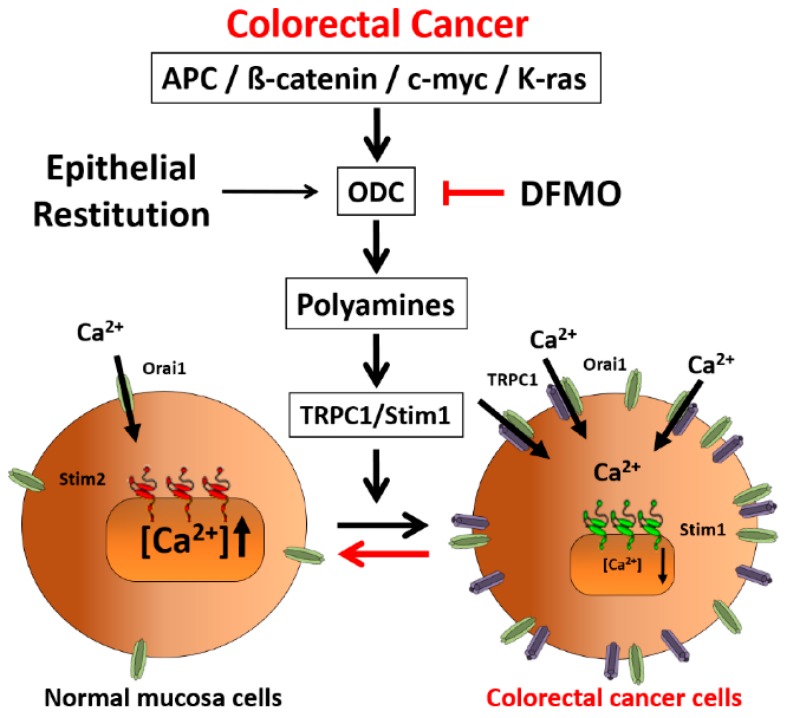
DFMO prevents colorectal cancer by reversing Ca^2+^ channel remodeling. Polyamines excess due to ODC overexpression associated to *adenomatous polyposis coli* (APC) gene dependent activation of *C-MYC and K-RAS* favors Ca^2+^ channel remodeling and cancer hallmarks. DFMO inhibits ODC and polyamine biosynthesis, thus, reversing Ca^2+^ remodeling in cancer and providing a mechanism for CRC chemoprevention.

In addition, DFMO treatment removes the non-selective component of SOCs that is solely present in CRC cells but not in normal colonic cells. As stated above, CRC cells display enhanced SOCE, and this increase correlates with enhanced cell proliferation and migration [22]. Consistently, DFMO treatment decreases both SOCE and cell proliferation in CRC cells. We reported previously that SOCs in normal colonic cells display only a Ca^2+^-selective inward current driven by Orai1 channels [22]. In contrast, CRC cells display larger currents with two components, a Ca^2+^ selective inward component and a non-selective, inward and outward component [22]. In contrast to normal cells, currents in CRC cells are driven by both Orai1 and TRPC1 channels, probably forming ion channel complexes specific of CRC cells. The I_SOC_ of HT29 cancer cells is significantly larger compared with the I_SOC_ from NCM460 normal colon cells. In addition, the I_SOC_ of HT29 does not display evident desensitization [31], likely contributing to sustained signaling in cancer cells. We report now that this TRPC1-dependent component observed only in CRC cells is selectively removed by treatment with DFMO and this effect is fully reversed by an excess of polyamine putrescine indicating that DFMO removes TRPC1 by preventing polyamine biosynthesis. Consistently, we show also that DFMO treatment decreases expression of both TRPC1 channels and STIM1, the sensor that gates Orai1/TRPC1 channel complexes present in CRC cells. Therefore, these results indicate that DFMO reverses the remodeling of SOCs in CRC and the mechanism involves changes in expression of TRPC1 and STIM1 (Figure 9).

Consistently with the changes in Isoc, we show also that DFMO treatment decreases expression of both TRPC1 channels and STIM1, the sensor that gates Orai1/TRPC1 channel complexes present in CRC cells. DFMO also inhibits expression of ORAI2. However, this isoform does not seem to play any role in SOCE or SOCs in HT29 cells [22]. In contrast, DFMO has no effect on STIM2 expression at the protein level, in spite of increasing expression at the messenger level. Interestingly, we reported that colon cancer HT29 cells display enhanced STIM1 expression along decreased STIM2 expression relative to normal NCM460 colonic cells [22], thus, resulting in enhanced STIM1 to STIM2 ratio in cancer cells. In spite that STIM2 protein expression does not change with DFMO, the ratio STIM1/STIM2 is indeed reduced by DFMO treatment consistently with reversal of the tumor phenotype. In addition, our results suggest that inhibition of TRPC1 by DFMO may occur at the transcriptional level while inhibition of STIM1 expression is post-transcriptional. Although further studies are needed to elucidate the DFMO mechanism, our results indicate that it reverses the remodeling of I_SOC_ in CRC and the mechanism involves changes in expression of TRPC1 and STIM1 (Figure 9).

Our results indicate not only the mechanism of CRC chemoprevention by polyamine synthesis inhibition with DFMO but also suggest that Ca^2+^ channel remodeling is an important step in CRC carcinogenesis and that this process is likely mediated by an excess of polyamine biosynthesis linked to ODC overexpression. It is well known that mutations in the *adenomatous polyposis coli* (APC) gene are responsible for *familial adenomatous polyposis* (FAP). In addition, APC play a rate-limiting role in most sporadic colorectal cancers, particularly by impinging on the WNT pathway [2]. In normal conditions, the WNT receptor is activated and transmits the signal intracellularly to the APC, glycogen synthase kinase-3β (GSK-3β) and β-catenin complex. GSK-3β phosphorylates β-catenin, marking it for proteosomal degradation [2]. In carcinogenesis, the WNT receptor may be activated, and transmission of the signal intracellularly may occur, but inactivation of β-catenin by GSK-3β does not. Cytoplasmic accumulation of β-catenin leads to its nuclear translocation and binding with its cognate partner TCF/LEF. This heterodimerization regulates genes through transcription, notably of C-MYC. MYC and K-RAS transcriptionally activate a number of polyamine metabolic genes, including ODC that converts ornithine to putrescine and other polyamines. Our present findings showing that polyamine synthesis inhibition reverses remodeling of SOCs from TRPC1/Orai1 channels characteristic of cancer cells to the classic Orai1 channels reported in normal cells suggest that channel remodeling is critical to the tumorigenic pathway induced by polyamines. This view is supported by the fact that the effect of DFMO on channel remodeling is reversed by putrescine. In addition, this possibility is also supported by a previous report showing that polyamines may regulate intestinal epithelial restitution through TRPC1-mediated Ca^2+^ signaling by altering the ratio of STIM1 to STIM2. As stated above, epithelial restitution is a physiological mechanism activated for tissue repair after the damage that involves the transient activation of cell migration and proliferation [11,12]. Polyamines are essential for epithelial restitution. As ODC is highly overexpressed in cancer, CRC can be envisioned as the result of activation of a program of chronic epithelial restitution process in the absence of damage.

DFMO is considered one of the best candidates for CRC chemoprevention. DFMO is particularly effective when combined with the NSAID sulindac. In fact, several clinical trials are testing the combination of sulindac and DFMO [6,7,8]. For example, this combination has been tested in people who had already had adenomas removed within the preceding 5 years. In that study, the development of high-risk adenomas and multiple adenomas was reduced by more than 90 percent in people who took the combination (*n* = 191 patients) compared with people who took the placebo (*n* = 184 patients) [6]. This preliminary study is the base of an ongoing phase III, Double-Blind Placebo-Controlled Trial of DFMO and sulindac to prevent the recurrence of high-risk adenomas and second primary colorectal cancers in patients with stage 0 to III colon cancer [9]. Interestingly, we have reported recently that sulindac is able to inactivate store-operated channels in colonic cells by mitochondria-dependent mechanism [13]. The mechanism is based on the ability of sulindac and other NSAIDs of acting as mild mitochondrial uncouplers that depolarize mitochondria partially, thus, limiting mitochondrial Ca^2+^ uptake. Consequently, Ca^2+^ entering through SOCE is not taken by mitochondria leading to Ca^2+^-dependent inactivation of SOCs [29,30]. This mechanism is highly efficient in limiting SOCE in normal cells but not as effective in CRC cells where TRPC1 channels do not inactivate. Here we show that DFMO treatment removes this component in CRC cells but not the Ca^2+^-selective component that is sensitive to sulindac. Consistently, we show that DFMO and sulindac are able to remove both components of SOCs in CRC cells, thus, abolishing SOCE in CRC cells and providing a mechanism for increased chemoprevention efficiency of the combination vs. therapy with a single compound.

In summary, our results suggest that Ca^2+^ channel remodeling that is associated to CRC and cancer hallmarks may be linked to excess polyamine biosynthesis associated with APC and/or K-ras mutations. In addition, they provide evidence that polyamine biosynthesis inhibition reverses Ca^2+^ channel remodeling in colon cancer cells, thus, providing a mechanistic basis for CRC chemoprevention. Finally, our results also provide evidence that the combination of both DFMO and sulindac inhibit both components of SOCs, the inward and outward components driven by both Orai1 and TRPC1 channels leading to efficient inhibition of SOCs and CRC chemoprevention.

## 4. Materials and Methods

### 4.1. Materials

HT29 cells were donated by Dr. J.C. Fernández-Checa (CSIC, Barcelona, Spain). Dulbecco’s Modified Eagle’s Medium (DMEM), Penicillin, streptomycin, L-glutamine, and fetal bovine serum is from Lonza (Basel, Switzerland). NCM460 were obtained after a material transfer agreement with INCELL Corporation (San Antonio, TX, USA). M3:10TM medium is from INCELL Corporation (San Antonio, TX, USA). Detachin is from Gelantis (San Diego, CA, USA). Fura-2/AM is from Invitrogen (Carlsbad, CA, USA). Thapsigargin is from Alomone Labs (Jerusalem, Israel). Primers were obtained from Thermo Scientific (Ulm, Germany). Antibodies against TRPC1, Orai1, Orai2, and STIM1 were from Alomone Labs (Jerusalem, Israel). Antibodies against STIM2 and ORAI3 were from Santa Cruz Biotechnology (Dallas, TX, USA). Anti-β-actin was from Abcam (Cambridge, UK)

### 4.2. Cell Culture

Cells were cultured in DMEM 1 g/L glucose (HT29) or in M3:10TM medium (NCM460) as reported previously [22] and supplemented with 1% Penicillin-Streptomycin, 1% L-glutamine and 10% fetal bovine serum. Cells were maintained in standard conditions (37 °C, 10% CO_2_) and subcultured once a week. All cells were used for experiments at passages 3 to 10.

### 4.3. Apoptosis Assays

Cell survival assay was performed by flow citometry using FITC Annexin V (BD Biosciences, Franklin Lakes, NJ, USA) and propidium iodide (Sigma, Steinheim, Germany). Cells were cultured with media containing solvent or DFMO for several days. Then cells were treated with solvent or 2 mM H_2_O_2_ for 150 min and then detached with trypsin-EDTA, centrifuged at 290 g and washed with cold PBS. Then, cells were suspended in binding buffer (0.1 M Hepes, pH 7.4, NaCl 1.4 M and 25 mM CaCl_2_) at a density of 1 × 10^6^ cells/ml. After that, 1 × 10^5^ cells were incubated with 5 µl of Annexin V and 10 µl of propidium iodide (50 µg/ml) for 15 min at room temperature in the dark. Cells were analyzed using Gallios Flow Cytometer (Beckman Coulter, Brea, CA, USA) and the results were processed with Kaluza Analysis Software (Beckman Coulter, Brea, CA, USA).

### 4.4. Cell Proliferation

Cells were plated in 6 well plates at a density of 10 × 10^5^ cells and incubated with supplemented DMEM or the same medium containing DFMO. Cells in wells were counted in triplicate at time 0 h and after 72, or 96 h using a hemocytometer. Cell viability was estimated using trypan blue staining.

### 4.5. Fluorescence Imaging of Cytosolic Ca^2+^

Cytosolic Ca^2+^ concentration ((Ca^2+^)_cyt_) was monitored as reported previously [22] by fluorescence imaging of cells using an inverted Zeiss Axiovert microscope equipped with a OrcaER Hamamatsu digital camera (Hamamatsu, Photonics, France). Cells were loaded with fura-2/AM (4 µM, 60 min) in external saline solution containing (in mM): 145 NaCl, 5 KCl, 1 CaCl_2_, 1 MgCl_2_, glucose 10, Hepes/Na 10 (pH 7.42). For SOCE, cells were washed twice and treated with CPA or thapsigargin (1 µM, 10 min) in the same medium except that it was devoid of Ca^2+^ and contained also 0.5 mM EGTA. Then cells were located in the stage of an inverted microscope and subjected to fluorescence imaging whereas continuously perfused with external medium at 37 °C. Cells are epi-illuminated alternately at 340 and 380 nm using band pass filters and light emitted above 520 nm at both excitation lights was filtered by the dichroic mirror, collected every 5 to 10 s with a 40×, 1.4 NA, oil objective.

### 4.6. Electrophysiological Recordings

I_SOC_ were recorded using a Port-a-patch planar patch-clamp system (Nanion Technologies, Munich, Germany) in the whole-cell, voltage-clamp configuration, at room temperature (20 ± 2 °C) as reported previously [22]. Cultured cells (3–5 days after plating) were detached with Detachin and suspended at a cell density of 1–5 × 10^6^ cells/mL in external recording solution containing (in mM): 145 NaCl, 2.8 KCl, 2 MgCl_2_, 10 CaCl_2_, 10 HEPES, 10 D-glucose (pH = 7.4). Suspended cells were placed on the NPC©1 chip surface, and the whole cell configuration was achieved. Internal recording solution containing (in mM): 50 CsCl, 60 CsF, 10 NaCl, 20 EGTA, 10 HEPES, 2 Na^+^-ATP (pH = 7.2, adjusted with CsOH) was deposited in recording chips, having resistances of 3–5 MΩ. I_SOC_ were activated with thapsigargin. I_SOC_ were assessed using voltage ramps (−100 to +100 mV in 200 ms) applied every 5 s, from a holding potential of 0 mV and acquired with an EPC-10 patch-clamp amplifier (HEKA). Immediately after the whole-cell configuration was established, the cell capacitance and the series resistances (<10 MΩ) were measured. During records, these two parameters were measured, and if exceed by 10% in respect to the initial value, the experiment was discontinued. Resting membrane potentials were estimated by reading the potential of the recorded cell immediately after rupturing the membrane in the current-clamp configuration. Leak currents were eliminated by subtracting the average of the first five ramp currents (obtained just after whole-cell configuration was reached) to all subsequent currents. Inward and outward current amplitude were measured at −80 mV and +80 mV, respectively. Data were normalized respect to cell capacitance. Liquid junction potential and capacitive currents were cancelled using the automatic compensation of the EPC-10. Data were filtered at 10 kHz and sampled at 5 kHz.

### 4.7. Quantitative RT-PCR

Total cellular RNA was isolated from cells using Trizol reagent (Invitrogen, Carlbads, CA, USA). Extracted RNA integrity was tested by electrophoresis on agarose gels, and the purity and concentration were determined by spectrophotometry. RNA was reverse transcribed using a High Capacity cDNA Reverse Transcription Kit (Applied Biosystems, Foster City, CA, USA) and the cDNA diluted before PCR amplification. Primers were designed with Primer-BLAST (28). Primer sequences used were reported previously [22]. Qualitative PCR was performed on a TGradient system (Biometra, Goettingen, Germany) using a Taq polymerase (Fermentas). The reactions protocol consisted in 3 min at 94 °C, 35 cycles of 1 min at 94 °C, 1 min at 57 °C, and 30 s at 72 °C and finished at 72 °C for 10 min. Real-time, quantitative PCR was performed using an SYBR green I detection in a LightCycler rapid thermal cycler (Roche, Mannheim, Germany). The PCR protocol started with 5 min at 95 °C followed by 45 cycles of 15 s at 95 °C, 20 s at 57 °C or at 60 °C and 5 s at 72 °C. *β-actin* was used as a housekeeping gene. The data were normalized by PCR analysis of *β-actin*. Melting curves were used to determine the specificity of PCR products [22]).

### 4.8. Western Blotting

Total protein was extracted from cells and used to quantify expression of TRPC1, STIM1, STIM2, Orai1, Orai2 and Orai3 as reported previously [22]. Whole-cell lysate was obtained using RIPA buffer (20 mM Tris–HCl, pH 7.8, 150 mM NaCl, 1% Triton X-100, 1% deoxycholic acid, 1 mM EDTA, 0.05% SDS) supplemented with the Halt™ Protease and Phosphatase Inhibitor Cocktail (100×) from ThermoFisher Scientific (ref #1861281) (Waltham, MC, USA). Protein concentrations were determined by a Bradford protein assay. Proteins were fractionated by SDS-PAGE, electroblotted onto PVDF membranes and probed with the antibodies at dilution 1/200 except the anti-*β-actin* that was used at dilution 1/5000. Antibodies were visualized by addition of goat anti-rabbit IgG or rabbit anti-mouse IgG. Detection was performed using Pierce ECL Western Blotting Substrate (Thermo Scientific) and VersaDoc Imaging System (BioRad, Munich, Germany). Quantification of protein expression was carried out using Quantity One software (BioRad, Munich, Germany).

### 4.9. Statistics

When only 2 means were compared, Student’s *t*-test was used. For more than 2 groups, the statistical significance of the data was assessed by ANOVA and compared using Bonferroni’s multiple comparison tests. Differences were considered significant at *p* < 0.05.

## 5. Conclusions

CRC is associated with enhanced and modified store-operated currents that include a non-selective current made of TRPC1. ODC, the limiting step in polyamine biosynthesis, is overexpressed in CRC cells. The ODC suicide inhibitor DFMO that prevents CRC, inhibits expression of TRPC1 and removes the non-selective component of store-operated currents, and this effect is reversed by polyamine putrescine. We conclude that polyamines may contribute to Ca^2+^ channel remodeling in CRC and DFMO may prevent CRC by reversing this remodeling.
